# Distinctive autophagy/mitophagy biomarker profiles in frontotemporal lobar degeneration and Alzheimer’s disease

**DOI:** 10.1186/s40478-025-01954-9

**Published:** 2025-02-20

**Authors:** Kateřina Veverová, Alžběta Katonová, Hana Horáková, Jan Laczó, Francesco Angelucci, Jakub Hort, Sofie Lautrup, Evandro Fei Fang, Martin Vyhnálek

**Affiliations:** 1 Memory Clinic, Department of Neurology, 2nd Faculty of Medicine, Charles University and Motol University Hospital, V Úvalu 84, Prague 5, 150 06 Czech Republic; 2 Department of Clinical Molecular Biology, University of Oslo and Akershus University Hospital, Lørenskog, 1478 Norway; 3 The Norwegian Centre on Healthy Ageing (NO-Age), Oslo, Norway

**Keywords:** Autophagy, Frontotemporal lobar degeneration, MAPT, Neurocognitive impairment, PINK1, TDP-43, TFEB

## Abstract

Maintaining cellular homeostasis by removing damaged and senescent mitochondria, a process termed mitophagy, is crucial in preventing Alzheimer’s disease (AD) and represents a promising therapeutic target. Our previous research revealed altered mitophagy biomarkers, such as increased CSF and serum PINK1 and serum BNIP3L and decreased serum TFEB levels, indicating impaired autophagy-lysosomal degradation in the AD continuum. However, the role of autophagy/mitophagy in frontotemporal lobar degeneration (FTLD) remains unclear. This study investigated the biomarkers of autophagy/mitophagy and lysosomal biogenesis (PINK1, ULK1, BNIP3L, and TFEB) in biofluids (CSF and serum) from 308 biomarker-defined individuals across the FTLD continuum (FTLD-dementia, *n* = 29; FTLD-MCI, *n* = 33) and compared them with those across the AD continuum (MCI-AD, *n* = 100; AD-dementia, *n* = 100) and cognitively unimpaired (CU) controls (*n* = 46) recruited from Czech Brain Aging Study. Additionally, we compared the mitophagy biomarkers across different FTLD clinical subtypes (frontal, semantic and nonfluent variant) with CU, and explored the association between mitophagy biomarkers and clinical phenotypes of FTLD (biomarkers of tau, biomarkers of neurodegeneration, cognition and ATN profile).

Our findings indicated a significantly lower CSF PINK1 and ULK1 levels in FTLD compared to AD, with FTLD dementia showing particularly low CSF PINK1 levels compared to AD-dementia. Conversely, CSF ULK1 levels were higher in FTLD-MCI compared to AD-dementia. Serum analyses revealed lower PINK1 and higher TFEB levels in FTLD dementia compared to AD dementia. This study provides compelling evidence of distinct alterations in autophagy/mitophagy biomarkers between FTLD and AD, indicating that these neurodegenerative diseases may affect the cellular waste disposal system through different pathways. This is the first study to explore mitophagy biomarkers in human CSF and serum in FTLD, opening avenues for further research and potential clinical applications.

## Introduction

In recent years, significant advances have been made in understanding the pathophysiology of neurodegenerative diseases. One of the important mechanisms affecting cellular function and leading to neurodegeneration is the impairment of mitophagy - the selective degradation of mitochondria by autophagy, targeting defective mitochondria following damage or stress [1]. Our study of human post-mortem brains from Alzheimer’s disease (AD) patients revealed that mitophagy declines with age and its capacity is reduced in AD patient-derived induced pluripotent stem cells (iPSC) neurons [2]. Animal models have also shown impaired mitophagy, which can be restored pharmacologically or genetically, reducing AD-related pathology and memory loss [2, 3]. These findings highlight the role of mitophagy in AD pathophysiology and underscore its potential as a promising therapeutic target.

Our previous clinical study identified alterations in mitophagy biomarkers across the AD continuum, with increased CSF and serum levels of PTEN-induced Kinase 1 (PINK1) and serum BCL2/adenovirus E1B 19 kDa protein-interacting protein 3-like (BNIP3L) (mitophagy activators) and decreased serum levels of transcription factor EB (TFEB) (a master regulator of lysosomal biogenesis) in AD dementia compared to controls [4]. These findings indicated an impairment in the final step of autophagy-lysosomal degradation in AD, as previously proposed [5].

Frontotemporal lobar degeneration (FTLD) is a clinically and neuropathologically heterogeneous group of neurodegenerative diseases affecting the frontal and temporal lobes [6, 7]. Clinically, the disease manifests either with predominant behavioral changes (behavioral variant frontotemporal dementia, bvFTD) or language impairment (semantic variant of primary progressive aphasia, svPPA or nonfluent variant of primary progressive aphasia, nvPPA). Roughly 95% of cases of FTLD are caused by tau and TAR DNA binding protein 43 (TDP-43) pathologies characterized by the deposition of 3R-tau repeat proteins and TDP-43, respectively [8, 9]. FTLD-tau is associated with the mutations in the *MAPT* gene encoding tau protein, and the disease clinically manifests mainly as bvFTD or nvPPA, while FTLD-TDP can be related to the mutations in the progranulin (*GRN*) or *C9orf72* genes, representing the majority of svPPA cases and an important cause of bvFTD [8]. Studies on animal models and cell cultures suggest impairment in the early mitophagy stage in mutations associated with *MAPT*, *GRN*, and *C9orf72* genes [10–13]. In addition, the C9orf72 mutation negatively influences the final stage of mitophagy as well [14–17]. Studies in human tissues and biofluids are scarce and provide contrasting results. Previously, elevated expression of TFEB and other lysosomal biogenesis proteins in FTLD brains was reported [18, 19], while others observed downregulation of lysosome-related genes in PBMCs from FTLD patients [20].

Unlike AD, the role of autophagy/mitophagy in the FTLD spectrum in various disease stages remains elusive. Based on a comparison between the established notion of impaired mitophagy in AD and the findings in FTLD, current research indicates that mitophagy plays distinct roles in the pathophysiology of AD and FTLD. To investigate this, we aimed to explore mitophagy biomarkers in the biofluids (CSF and serum) of biomarker-defined individuals in the FTLD continuum (FTLD-dementia, *n* = 23, FTLD-MCI, *n* = 30) and compare them to individuals in the AD continuum (AD-MCI, *n* = 100, AD-dementia, *n* = 100) and cognitively unimpaired (CU) controls (*n* = 46). Additionally, we aimed to compare mitophagy biomarkers across the clinical spectrum of FTLD individuals (bvFTD, nvPPA, svPPA) with CU individuals and to characterize the specific alterations in mitophagy protein expression associated with different clinical presentations of FTLD. To further uncover the role of mitophagy in FTLD, we aimed to analyze the relationship between mitophagy markers and other standard neurodegenerative markers– neurofilament light chain (NfL), phosphorylated tau protein 181 (P-tau181) and total tau protein (T-tau) levels.

In our study, we found a significant decrease in PINK1 levels in FTLD and an increase in AD compared to CU. In contrast, ULK1 and TFEB were increased in FTLD compared to AD. To the best of our knowledge, this is the first study that explored the potential of mitophagy proteins as biomarkers in human CSF and serum in individuals with FTLD.

## Materials and methods

### Participants

This study included 308 biomarker-defined participants recruited from the Czech Brain Aging Study (CBAS) cohort, the CBAS plus cohort at the Memory Clinic of Charles University/Motol University Hospital, and the Department of Neurology, Motol University Hospital in Prague, Czech Republic. All study participants signed an informed consent form approved by the local ethics committee (number EK218/20) [19]. All participants included in the study were White and of Czech nationality. Participants with cognitive deficit (*n* = 262) were referred to the Memory Clinic by general practitioners or neurologists for cognitive difficulties reported by themselves or their informants. Cognitively unimpaired (CU) older adult participants (*n* = 46) had previously undergone lumbar puncture that was negative for pathological conditions.

All study participants were subjected to clinical evaluation, including routine blood tests, cognitive assessment, brain magnetic resonance imaging (MRI) and quantitative assessment of Aß in the brain. The majority of participants (*n* = 218) underwent lumbar puncture to donate CSF, which was analyzed for amyloid beta 42 (Aβ42), phosphorylated tau protein 181 (P-tau181) and total tau protein (T-tau) levels; patients who did not donate CSF (*n* = 24) underwent PET imaging to assess Aβ load.

#### Participants with FTLD

1a The cohort of participants with FTLD dementia (*n* = 29) and MCI due to FTLD (FTLD-MCI) (*n* = 33) comprised all major FTLD clinical subtypes. Both behavioral and language variants were included. Patients with behavioral variant of frontotemporal dementia (bvFTD) met the revised diagnostic criteria by Rascovsky [6]. Patients with language variants fulfilled the clinical criteria for a nonfluent variant of primary progressive aphasia (nvPPA) or semantic variant of a primary progressive aphasia (svPPA) [7]. In total, we included 25 individuals with svPPA (FTLD dementia [*n* = 12], FTLD-MCI [*n* = 13]), which is most often associated with TDP-43 pathology (FTLD-TDP), 10 individuals with nvPPA of FTLD (FTLD dementia [*n* = 4], FTLD-MCI [*n* = 6]), which is mainly associated with tau pathology (FTLD-tau), and 27 individuals with bvFTD (FTLD dementia [*n* = 13], FTLD-MCI [*n* = 14]), which is pathologically both FTLD-tau/FTLD-TDP [10]. Two patients from bvFTD subsequently manifested corticobasal syndrome and one patient developed mild parkinsonian syndrom.

In the lack of standardized FTLD-MCI criteria, the classification into clinical stages (dementia or mild cognitive impairment) was based on the level of functional impairment, following Petersen’s criteria: patients with cognitive impairment but preserved functional abilities, thereby not meeting the criteria for dementia, were classified as MCI [11]. Conversely, patients with cognitive impairment causing significant impairment in activities of daily living were categorized as having dementia. Concepts of MCI and FTLD, particularly bvFTD, may present potential conflicts. While MCI emphasizes preserved functional independence, probable bvFTD requires significant functional decline. Our FTLD-MCI individuals met the criteria for possible bvFTD as defined by Rascovsky et al. [6], which does not require significant functional decline. This approach allowed for the identification of early stages of FTLD where cognitive and behavioral changes are evident but independence in daily activities is largely preserved.

Additionally, patients with primary progressive aphasia (PPA) variants, including svPPA and nvPPA, were categorized as MCI if their language impairments were isolated, and daily functioning remained relatively intact. This approach aligns with the clinical presentation of these PPA subtypes in their early stages, as defined by Gorno-Tempini et al. [7], where language deficits precede significant functional or cognitive decline.

To reduce the potential impact of mixed pathologies on our results, only patients with negative AD amyloid biomarkers (Aβ42 > 620 pg/mL) or negative amyloid PET were included in the FTLD group.

#### Participants with AD

2a Participants with AD dementia (*n* = 100) met the clinical criteria for a high likelihood of AD dementia, including evidence of AD pathophysiology, progressive impairment in at least two cognitive domains (i.e.,≥1.5 standard deviations [SD] lower memory test score than the age- and education-adjusted norms as well as similarly low score in at least one non-memory cognitive test) and significant impairment in activities of daily living [12]. The participants had CSF positive for AD biomarkers (reduced Aβ42 and elevated P-tau181 [< 620 pg/mL and > 61 pg/mL, respectively] [13]) (*n* = 93) and/or were positive for Aβ based on 18F-flutemetamol PET scan (*n* = 9). In patients who did not undergo CSF withdrawal, neuronal injury was determined based on visual rating of the hippocampus [14].

2b Participants with mild cognitive impairment due to AD (MCI-AD) (*n* = 100) met the clinical criteria for a high likelihood of aMCI due to AD including memory complaints, evidence of memory impairment (i.e., ≥ 1.5 SDs lower score than the age- and education-adjusted norms in any memory test), but largely intact activities of daily living and an absence of dementia [15]. The participants had CSF positive for AD biomarkers (reduced Aβ42 and elevated P-tau181 [< 620 pg/mL and > 61 pg/mL, respectively] [13]) (*n* = 90) and/or were positive for Aβ based on PET imaging (*n* = 8). In patients who did not undergo CSF withdrawal, the neuronal injury was determined based on a visual rating of the hippocampus [14]. As expected, MCI-AD scored lower than CU participants and higher than AD dementia patients in all cognitive tests (ps < 0.05).

#### CU participants (*n* = 46) met one of two sets of criteria

3a Participants referred by the Department of Neurology met the following criteria: underwent lumbar puncture to exclude inflammatory disorders (e.g., facial palsy, headache, back pain), were negative for pathological and inflammatory markers in CSF and blood (*n* = 39), did not report cognitive complaints, demonstrated cognitive performance within the age- and education-adjusted normal range. In addition, they had no evidence of hippocampal atrophy on MRI and had normal results of AD biomarkers in CSF.

3b Patients followed in the CBAS study for subjective cognitive decline (SCD) (*n* = 7). These SCD controls reported cognitive complaints that motivated them to seek medical help, but they did not have any impairment in activities of daily living and demonstrated cognitive performance within the normal age- and education-adjusted range [16]. In addition, they had no evidence of hippocampal atrophy on MRI and had normal results of AD biomarkers in CSF or on PET.

Demographic data are shown in Table 1.

**Table 1:** Characteristic of study participants

	Memory Clinic Cohort				CU Controls
	FTLD dementia	FTLD-MCI	AD dementia	AD-MCI	
**Demographic characteristics**	**n = 29**	**n = 33**	**n = 100**	**n = 100**	**n = 46**
Age, years (SD)	65.0 (7.7)	66.1 (7.3)	70.3 (8.6)^***, ++^	72.5 (6.9)^***, +++, ††^	63.8 (9.4)
Female, n (%)	11 (37.9)^**^	14 (42.4)^*^	64 (64.0) ^+, †^	57 (57.0)	33 (71.7)
Education, years (SD)	14.3 (2.6)	15.1 (2.6)	14.0 (2.9)^*^	14.7 (3.1)	15.7 (2.8)
APOE ε4 positive, n(%)	3 (10.3)	6 (18.1)	52 (52.0)^**, †††, +++^	57 (57.0)^**, †††, +++^	8 (17.4)
MMSE score (SD)	20.6 (5.2)^***^	25.9 (4.0)^**, +++^	18.5 (4.2) ^***, †††^	24.9 (3.0)^***, +++,×××^	29.0 (1.2)
GDS-15	4.1 (3.0)	3.6 (2.3)	3.6 (3.1)	3.0 (2.4)	2.2 (2.3)
BAI	6.4 (8.1)	9.9 (7.5)	9.1 (7.5)	8.6 (7.6)	9.3 (6.6)

Data are presented as n (%) and mean (SD) unless otherwise specified. *P* values are comparisons using Tukey post hoc tests (one-way analysis of variance was used to test the main between group differences)

Abbreviations: FTLD, frontotemporal lobar degeneration; MCI, mild cognitive impairment; AD, Alzheimer’s disease; CU, cognitively unimpaired; APOE, apolipoprotein E; MMSE, Mini Mental State Examination; GDS-15, Geriatric Depression Scale, 15-item version; BAI, Beck Anxiety Inventory

*Missing genotype data in *n* = 71 (8 FTLD-dementia, 6 FTLD-MCI, 21 AD-dementia, 15 AD-MCI, 21 CU)

For *p* values indicating the level of significance compared with control group: *p <.05; **p <.01; **, *p* <.001***; compared with FTLD dementia group: + *p* <.05; ++ *p* <.01; +++ *p* <.001; compared with MCI due to FTLD group: † *p* <.05; †† *p* <.01; ††† *p* <.001; compared with AD dementia group: × *p* <.05; ×× *p* <.01; ××× *p* <.001

Besides the clinical classification, the patients (AD and FTLD groups) undergoing CSF withdrawal (*n* = 216) were also classified according to the AT(N) criteria framework. To define the AT(N) status, we used CSF Aβ42 as “A”, CSF P-tau181 as “T”, and CSF T-tau as “N” [17]. These biomarkers were dichotomized as normal (-) or abnormal (+), and the patients were divided into six groups as follows: A-T-N- (normal AD biomarkers, *n* = 32), A-T + N-/A-T-N+ (non-AD pathologic change, *n* = 16), A-T + N+ (non-AD pathologic change, *n* = 17), A + T-N- (amyloid pathology, *n* = 32), A + T + N- (AD pathology, *n* = 30), A + T + N+ (AD pathology with neurodegeneration, *n* = 89) [25]. Individuals with AT(N) profiles fulfilling the category of AD pathology with concomitant non-AD pathological change A + T-N+ (*n* = 2) were excluded from the analyses for the low number of subjects and possible mixed pathology.

### Exclusion criteria

Participants with moderate to severe white matter vascular lesions on MRI (Fazekas score > 2 points) or the presence or history of any primary neurological or psychiatric disorders that could cause cognitive or mitochondrial dysfunction (e.g., major depressive disorder, psychosis, substance abuse, Lewy body dementia, Parkinson’s disease or parkinsonian syndromes not belonging to the FTLD spectrum, multiple sclerosis) were excluded from the study. Additionally, patients with evidence of cancer, diabetes, renal failure (defined as a GFR < 60 mL/min/1.73 m² or chronic kidney damage) or cardiac failure were excluded based on a combination of medical records, blood test results, a pharmacotherapy review, and thorough evaluation by the treating physicians.

### CSF and blood collection and processing

For both blood and CSF samples, a standardized protocol was followed. Blood was drawn through venipuncture and allowed to clot for 15 min at room temperature. Within 30 min of collection, it was centrifuged at 1700 x g for 5 min at 20 °C. Separated serum was then divided into 0.5 mL aliquots in polypropylene tubes and stored at -80 °C until analysis.

CSF was collected into 8-mL polypropylene tubes via lumbar puncture in a supine position at L3/L4 or L4/L5. After gentle mixing, it was centrifuged at 1700 x g for 5 min at 20 °C within 30 min of collection. Aliquots of 0.5 mL were stored in polypropylene tubes at -80 °C until analysis. Finally, both serum and CSF samples were thawed and vortexed for 15 s before biomarker analysis.

### Immunological assays

CSF biomarkers (Aβ42, Aβ40, T-tau, P-tau181, and NfL) were quantified by commercially available enzyme-linked immunosorbent assay (ELISA) kits (EUROIMMUN, Lübeck, Germany for Aβ42, Aβ40, T-tau, and P-tau181; cat. no. EQ 6521-9601-L for Aβ42, cat. no.

EQ 6511-9601-L for Aβ40, cat. no. EQ 6531-9601-L for T-tau, and cat. no. EQ 6591-9601-L for P-tau181; UmanDiagnostics, Umeå, Sweden; cat.no. 10-7001CE for NfL).

Protein levels of mitophagy biomarkers ULK1 and PINK1 (in both CSF and serum) and BNIP3L and TFEB (in serum only) were determined using commercially available ELISA kits (FineTest, Wuhan, Hubei, China; cat. no. EH4191 for ULK1 and cat. no. EH6731 for BNIP3L; MyBioSource, San Diego, California, USA; cat. no. MBS7607221 for PINK1 and MBS7612687 for TFEB).

All assays were performed according to the manufacturer’s instructions. Both serum and CSF samples were analyzed in duplicate to ensure analytical precision and reduce potential measurement error. At the end of each assay, absorbance was measured at 450 nm using a microplate reader (Dynex Technologies, Virginia, USA). Protein concentrations were then calculated by comparison to a standard curve. Reported intra-assay coefficients of variation (CV%) remained below 8% for all biomarkers and inter-assay CV% was below 10% for all biomarkers.

### Neuropsychological assessment

The comprehensive neuropsychological assessment covered the following domains: (a) global cognitive function measured with Mini-Mental State Examination (MMSE) [18]; (b) attention and working memory measured with the Forward and Backward Digit Span subtests (DS-F, DS-B, respectively), adaptation from the Uniform Data Set (UDS-cz 2.0 [19]), and the Trail Making Test (TMT) A [20]; (c) memory measured with the Logical Memory (LM) immediate and delayed recall, an adaptation from the UDS-cz 2.0 [19]; (d) language measured with the Boston Naming Test (BNT-30), 30-items odd version [21], and semantic verbal fluency (S-VF, animals) [22]; (e) executive function measured with TMT B [20], and phonemic verbal fluency (P-VF, Czech version with letters N, K, P) [22]; and (f) visuospatial function measured with the Rey-Osterrieth Complex Figure Task (ROCFT) —the copy condition, and the Clock Drawing Test (CDT) [23]. To evaluate anxiety and depressive symptoms, the self-reported Geriatric Depression Scale, a 15-item version (GDS-15; [24]), and the Beck Anxiety Inventory (BAI; [25]) were administered. Mean cognitive performance values (± SD) are listed for each patient subgroup in Table 2.

**Table 2:** Neuropsychological characteristics of study participants

	Memory Clinic Cohort				CU Controls
	FTLD dementia	FTLD-MCI	AD dementia	AD-MCI	
**Neuropsychological tests**					
DS-F	7.7 (1.9)***	6.5 (2.6)*	7.2 (2.2)***	8.4 (2.0)^++, ××^	9.4 (2.3)
DS-B	3.6 (1.9)***	5.0 (2.0)**	3.7 (1.7)***^, †^	5.1 (1.7)***^, ++, ×××^	6.6 (1.7)
TMT A	80.5 (55.5)**	65.0 (34.9)	107.1 (56.0)*** ^, †††^	64.4 (32.3)** ^, ×××^	37.3 (12.9)
TMT B	245.8 (79.1)***	186.3 (87.9)***	272.3 (61.9)***^, †††^	198.8 (86.5)***^, ×××^	104.4 (60.1)
P-VF	16.1 (12.0)***	25.8 (14.3)***	25.1 (10.3)***^, +^	37.2 (13.4)**^, +++, †††, ×××^	47.0 (12.8)
S-VF	8.3 (4.4)***	12.5 (7.1)***	11.6 (5.2)***	16.5 (5.3)***^, +++, †, ×××^	27.1 (6.1)
BNT	13.1 (6.1)***	7.7 (5.3)***^, ++^	9.9 (5.0)***	6.2 (4.5)***^, +++, ×××^	1.7 (1.7)
CDT	12.0 (3.2)**	13.9 (2.1)	9.4 (3.9)***^, ++, †††^	13.0 (2.4)**^, ×××^	15.5 (0.7)
ROCF-C	21.1 (10.5)***	27.2 (6.0)	18.0 (10.5)***	25.3 (6.6)*^, ×××^	30.1 (3.3)
LM-IR	5.5 (3.6)***	9.5 (4.5)***^, +^	4.6 (3.4)***^, †††^	8.2 (4.2)***^, ×××^	17.7 (4.5)
LM-DR^a^	3.1 (3.3)***	7.3 (5.0)***^, ++^	1.6 (2.4)***^, †††^	3.7 (4.6)***^, ††, ×^	15.7 (5.3)

Data are presented as mean (SD). *P* values are comparisons using Tukey post hoc tests (one-way analysis of variance was used to test the main between group differences)

DS-F, Digit Span Forward; DS-B, Digit Span Backward; TMT, Trail Making Test; P-VF, phonemic verbal fluency (letters N, K, P); S-VF, semantic verbal fluency (animals); BNT, Boston Naming Test (30 odd-items version), number of errors; CDT, Clock Drawing Test; ROCF-C, Rey-Osterrieth Complex Figure, copy condition; LM-IR, Logical Memory Story I, immediate recall; LM-DR, Logical Memory Story I, delayed recall

For *p* values indicating the level of significance compared with control group: *p <.05; **p <.01; **, *p* <.001***; compared with FTLD dementia group: + *p* <.05; ++ *p* <.01; +++ *p* <.001; compared with MCI due to FTLD group: † *p* <.05; †† *p* <.01; ††† *p* <.001; compared with AD dementia group: × *p* <.05; ×× *p* <.01; ××× *p* <.001

### MRI acquisition and analysis

MRI images were acquired on a 1.5T scanner (Siemens, Erlangen, Germany) using T1-weighted three-dimensional high-resolution magnetization-prepared rapid acquisition with gradient echo sequence using the following parameters: TR/TE/TI = 2000/3.08/1100 ms, flip angle 15°, 192 continuous partitions, slice thickness 1.0 mm, and in-plane resolution 1 mm [26]. All images were inspected visually by a neuroradiologist in a blinded manner. Patients whose MRI data showed evidence of tumor, cortical infarct, hydrocephalus, or other major anatomical variation were excluded from the study. Freesurfer automated suite (v7.1.0, http://surfer.nmr.mgh.harvard.edu) was used to derive regional cortical thickness [27], subcortical areas and volumes [28], as well as total hippocampal volume, entorhinal cortical thickness, parahippocampal cortical thickness, total gray matter volume, and ventricular volumes. Brain area and volume measurements were normalized to the estimated total intracranial volume (eTIV) [29], as follows: Vol_adj_=Vol_raw_ − β(TIV_raw_−TIV_mean_); regional thickness measurements were not eTIV-adjusted [30].

### Statistical analysis

All data were standardized to z-scores. Data with non-normal distribution were log-transformed (mitophagy markers, biomarkers of tau and neurodegeneration) prior to transformation to z-scores.

Between-group differences in demographical characteristics were evaluated using parametric one-way analysis of variance (ANOVA) with Tukey post hoc tests for continuous variables (age, years of education, MMSE, GDS and BAI score) and chi-square tests for dichotomous variables (sex, APOE ε4 status).

Between-group differences in mitophagy markers were evaluated using parametric one-way analysis of covariance (ANCOVA), with Tukey post hoc tests. Each ANCOVA model included the mean value of the mitophagy biomarker level as the outcome, the study group as a between-subject factor, and covariates of age and sex.

The relationships between mitophagy markers (PINK1, ULK1, BNIP3L, TFEB), biomarkers of tau (P-tau181, T-tau), biomarkers of neurodegeneration (NfL) and AT(N) profiles were evaluated using partial Pearson correlations adjusted for age and sex. Pearson correlation adjusted for age, sex, and years of education were used to establish the relationship between mitophagy markers (PINK1, ULK1, BNIP3L, TFEB) with the cognitive composite domain (attention and working memory, memory, executive function, language, and visuospatial function). Holm-Bonferroni correction was used to adjust for multiple comparisons.

Cognitive domains were expressed as composite domain z-scores, calculated as the average of z-scores for each of the tests within the specific cognitive domain. The z-scores for TMT A and B, and BNT-30 were multiplied by negative ones to express the values in the same direction as the other neuropsychological values. The maximum times for completion of the TMT A and B were 180 s and 300 s, respectively, and those who were unable to complete the tests were assigned a score of as 181 s and 301 s, respectively.

p-values < 0.05 (*), 0.01 (**), and 0.001 (***) were considered statistically significant. Analyses were performed using the R statistical language environment [31].

## Results

### Changes of mitophagy proteins in CSF and serum from FTLD-MCI, FTLD dementia, AD-MCI, AD dementia and CU

Using commercially available ELISA kits, PINK1 and ULK1 were detected in CSF, while BNIP3L and TFEB were not. This could reflect that the antigen concentration in CSF was below the level of detection (all tested with commercially available kits). PINK1, ULK1, BNIP3L, and TFEB were detected in blood serum using commercially available ELISA kits.

**Fig. 1:**
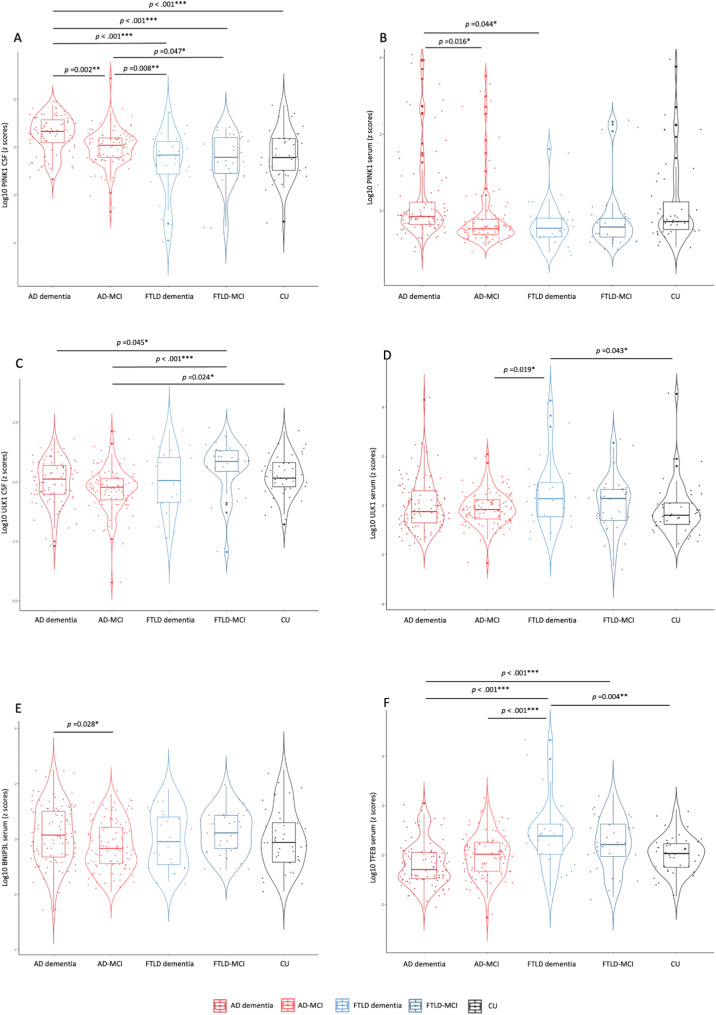
Changes of mitophagy proteins in CSF and serum in clinical cohort. Violin plots of ANCOVA analyses (A–F) of levels of mitophagy biomarkers PINK1, ULK1, BNIP3L, and TFEB in biomarker-defined individuals. (**A**) CSF PINK1 showed lower levels in both FTLD dementia and FTLD-MCI compared to AD dementia and AD-MCI groups. It also showed higher levels in AD dementia compared to AD-MCI and CU. (**B**) Serum PINK1 showed lower levels in FTLD dementia compared to AD dementia and higher levels in AD dementia compared to AD-MCI. (**C**) CSF ULK1 showed higher levels in FTLD-MCI compared to AD dementia and AD-MCI groups. It also showed lower levels in AD-MCI compared to CU. (**D**) Serum ULK1 showed higher levels in FTLD dementia compared to AD-MCI and CU. (**E**) Serum BNIP3L showed higher levels in AD dementia compared to AD-MCI. (**F**) Serum TFEB showed higher levels in FTLD dementia compared to AD dementia, AD-MCI and CU groups. It also showed higher levels in FTLD-MCI compared to AD dementia Notes: Data were adjusted for sex and age. *** = *p* <.001, ** = *p* <.01, * = *p* <.05. Abbreviations: AD, Alzheimer’s disease; FTLD, frontotemporal lobar degeneration; MCI, mild cognitive impairment; CSF, cerebrospinal fluid; CU, cognitively unimpaired

In CSF, we identified a significant difference among study subgroups in PINK1 levels (F [4] = 14.98, *p* <.001, $\:\eta\:$^2^ = 0.18). Post hoc analyses revealed that FTLD dementia individuals had significantly lower PINK1 CSF levels (0.94 $\:\pm\:$ 0.30 ng/mL) compared to AD dementia (1.26 $\:\pm\:$ 0.24 ng/mL) (*p* <.001) and AD-MCI (1.09 $\:\pm\:$ 0.23 ng/mL) (*p* =.009). Similarly, individuals with FTLD-MCI (0.96 $\:\pm\:$ 0.24 ng/mL) showed lower levels compared to AD dementia (*p* <.001) and AD-MCI individuals (*p* =.047). Additionally, AD dementia individuals showed increased levels of CSF PINK1 compared to AD-MCI (*p* =.002) and controls (1.02 $\:\pm\:$ 0.28 ng/mL) (*p* <.001) (Fig. 1A). Similar results were found in serum (F [4] = 3.65, *p* =.007, $\:\eta\:$^2^ = 0.06), with lower PINK1 in FTLD dementia (1.87 $\:\pm\:$ 1.54 ng/mL) than in AD dementia individuals (6.18 $\:\pm\:$ 12.86 ng/mL) (*p* =.044). We also found higher serum PINK1 levels in AD dementia compared to AD-MCI (3.12 $\:\pm\:$ 6.55 ng/mL) (*p* =.016) (Fig. 1B).

Likewise, CSF ULK1 levels in FTLD-MCI (187.4 $\:\pm\:$ 69.1 pg/mL) were higher compared to both AD-dementia (147.5 $\:\pm\:$ 53.1 pg/mL) and AD-MCI individuals (129.9 $\:\pm\:$ 47.8 pg/mL) (*p* =.045, *p* <.001, respectively) (F [4] = 4.93, *p* <.001, $\:\eta\:$^2^ = 0.08). AD-MCI had lower CSF ULK1 levels compared to CU (162.5 $\:\pm\:$ 56.6 pg/mL) (*p* =.024) (Fig. 1C). Serum ULK1 levels also differed among the study subgroups (F [4] = 2.65, *p* =.034, $\:\eta\:$^2^ = 0.04), with higher levels in FTLD dementia (177.8 $\:\pm\:$ 236.1 pg/mL) compared to AD-MCI (83.6 $\:\pm\:$ 39.6 pg/mL) (*p* =.019) and CU (108.6 $\:\pm\:$ 190.6 pg/mL) (*p* =.043) (Fig. 1D).

Serum BNIP3L differed among the patient subgroups (F [4] = 2.89, *p =*.023, $\:\eta\:$^2^ = 0.04), but post hoc analysis did not show any differences regarding FTLD subgroups. We only found higher BNIP3L levels in AD dementia (2.90 $\:\pm\:$ 2.07 ng/mL) compared to AD-MCI (2.06 $\:\pm\:$ 1.23 ng/mL) (*p* =.027) (Fig. 1E).

In contrast, serum TFEB levels were elevated in FTLD dementia (1185.9 $\:\pm\:$ 999.0 pg/mL) compared to AD dementia (634.8 $\:\pm\:$ 275.7 pg/mL) (*p*<.001), AD-MCI (720.9 $\:\pm\:$ 276.7 pg/mL) (*p*<.001) and CU individuals (728.1 $\:\pm\:$ 233.3 pg/mL) (*p* <.004). Also, FTLD-MCI (899.1 $\:\pm\:$ 399.4 pg/mL) showed higher levels of serum TFEB compared to AD dementia (*p* <.001) (F [4] = 11.15, *p* <.001, $\:\eta\:$^2^ = 0.13). (Fig. 1F). These results are summarized in Table 3.

**Table 3:** Mitophagy and AD biomarkers in CSF and serum

	Memory Clinic Cohort					CU Controls	
	FTLD dementia	FTLD-MCI	AD dementia	AD-MCI			
**Mitophagy biomarkers**	**n = 23**	**n = 30**	**n = 100**	**n = 100**		**n = 46**	
CSF ULK1, pg/mL (SD)	155.5 (84.5)	187.4 (69.1)	147.5 (53.1)	129.9 (47.8)^*, †††^		162.5 (56.6)	
Serum ULK1, pg/mL (SD)	177.8 (236.1)^*^	114.8 (89.9)	107.1 (121.5)	83.6 (39.6)^+^		108.6 (190.6)	
CSF PINK1, ng/mL (SD)	0.94 (0.30)	0.96 (0.24)	1.26 (0.24)^***, †††, +++^	1.09 (0.23)^×××^		1.02 (0.28)	
Serum PINK1, ng/mL (SD)	1.87 (1.54)	3.42 (4.97)	6.18 (12.86)	3.12 (6.55)^×^		4.80 (9.62)	
Serum BNIP3L, ng/mL (SD)	2.68 (1.69)	2.72 (1.43)	2.90 (2.07)	2.06 (1.23)^×^		2.70 (2.19)	
Serum TFEB, pg/mL (SD)	1185.9 (999.0)^**^	899.1 (399.4)^×××^	634.8 (275.7)^+++^	720.9 (276.7)^+++^		728.1 (233.3)	
**AD biomarkers**							
CSF Aβ42/ Aβ40 ratio	0.09 (0.03)	0.08 (0.01)	0.04 (0.01)^***, †††, +++^	0.04 (0.02)^***, †††, +++^		0.09 (0.02)	
CSF Aβ42, pg/mL	1018.4 (401.0)	1035.0 (393.3)	497.4 (204.8)^***, †††, +++^	484.2 (166.4)^***, †††, +++^		1157.7 (440.5)	
CSF T-tau, pg/mL	408.8 (268.5)^**^	305.1 (147.0)^*^	653.3 (451.1)^***, †††, ++^	565.1 (316.8)^***, †††^		219.6 (109.7)	
CSF P-tau181, pg/mL	63.2 (46.2)^**^	44.5 (16.9)	105.5 (67.0)^***, †††, ++^	95.5 (57.6)^***, †††, +^		36.8 (17.2)	
CSF NfL pg/mL	3026.1 (2303.3)^***^	2596.3 (1587.1)^***^	1571.2 (979.8)^***, ††, +++^	1304.2 (864.1)^***, †††, +++^		666.1 (455.3)	
Serum NfL pg/mL	69.9 (59.0)^***^	42.3 (33.9)^**^	38.9 (20.7)^***^	35.8 (31.2)^***, +^		16.3 (6.3)	
CSF Ng, pg/mL	200.8 (73.2)	172.4 (61.7)	265.2 (108.3)^*, †^	253.8 (101.6)^†^		182.5 (70.3)	
SIMOA NfL	50.4 (44.4)^***^	40.9 (20.3)^***^	57.6 (164.1)^***^	37.2 (26.1)^***^		17.7 (10.1)	
SIMOA GFAP	267.5 (216.1)	187.4 (90.0)	679.4 (1234.9)^***, †††, +++^	503.8 (267.9)^***, †††, +++^		191.2 (81.3)	

Data are presented as N(%) and mean (SD) unless otherwise specified

*P* values are comparisons using Tukey post hoc tests (one-way analysis of covariance was used to test the main between group differences), data were log-transformed

Abbreviations: FTLD, frontotemporal lobar degeneration; MCI, mild cognitive impairment; AD, Alzheimer’s disease; CU, cognitively unimpaired; CSF, cerebrospinal fluid; NfL, neurofilament light; Ng, neurogranin

For *p* values indicating the level of significance compared with control group: *p <.05; **p <.01; ***p <.001; compared with FTLD dementia group: + *p* <.05; ++ *p* <.01; +++ *p* <.001; compared with MCI due to FTLD group: † *p* <.05; †† *p* <.01; ††† *p* <.001; compared with AD dementia group: × *p* <.05; ×× *p* <.01; ××× *p* <.001

### Changes of mitophagy proteins in CSF and serum in FTLD clinical subtypes (bvFTLD, svPPA, nvPPA)

To assess whether the differences between the study groups, particularly FTLD vs. CU, were driven by specific FTLD clinical subtypes, we compared the mitophagy biomarker levels between bvFTLD, svPPA, nvPPA, and CU.

**Fig. 2:**
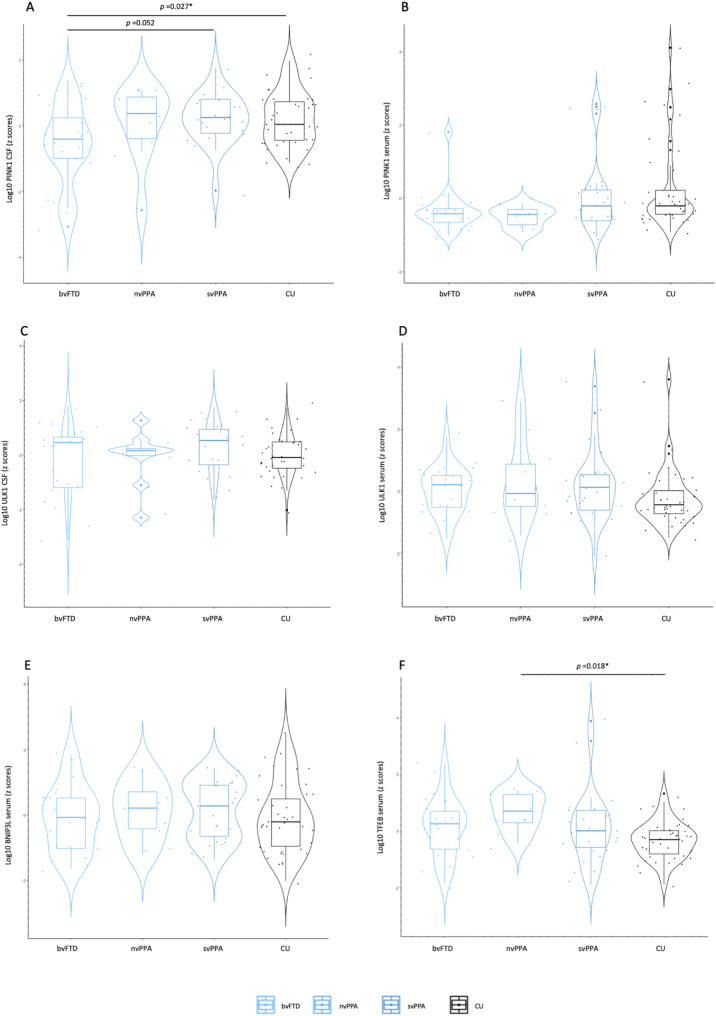
Changes of mitophagy proteins in the clinical spectrum of FTLD. Violin plots of ANCOVA analyses (A-F) of levels of mitophagy biomarkers PINK1, ULK1, BNIP3L, and TFEB in the clinical spectrum of FTLD. (A) CSF PINK1 showed lower levels in bvFTD compared to CU and was almost significantly lower in bvFTD compared to svPPA. (B) Serum PINK1 levels did not show any differences among the groups. (C) CSF ULK1 levels did not show any differences among the groups. (D) Serum ULK1 levels did not show any differences among the groups. (E) Serum BNIP3L levels did not show any differences among the groups. (F) Serum TFEB showed higher levels in nvPPA compared to CU Notes: Data were adjusted for sex and age. ***p <.001; **p <.01; *p <.05. Abbreviations: AD, Alzheimer’s disease; FTLD, frontotemporal lobar degeneration; MCI, mild cognitive impairment; CSF, cerebrospinal fluid; CU, cognitively unimpaired

Our analyses showed that CSF PINK1 levels differed among the FTLD subtypes (F [3] = 3.19, *p* =.028, η2 = 0.10). Post hoc tests revealed that bvFTD (0.85 $\:\pm\:$ 0.29 ng/mL) had lower CSF PINK1 levels compared to CU controls (1.02 $\:\pm\:$ 0.28 ng/mL) (*p* =.027) and almost reached significance with lower levels compared to svPPA (1.03 $\:\pm\:$ 0.23 ng/mL) (*p* =.052). No other significant differences were observed (Fig. 2A). Serum PINK1 levels did not differ between FTLD clinical subtypes and CU individuals (F [3] = 2.07, *p* =.111, η2 = 0.07) (Fig. 2B), nor did CSF ULK1 (F [3] = 1.05, *p* =.377, η2 = 0.04) (Fig. 2C), serum ULK1 (F [3] = 1.31, *p* =.275, η2 = 0.04) (Fig. 2D) or serum BNIP3L levels (F [3] = 0.81, *p* =.494, η2 = 0. 01) (Fig. 2E). Serum TFEB levels differed between the FTLD subtypes (F [3] = 3.58, *p* =.017, η2 = 0.11), with nvPPA (1163.08 $\:\pm\:$ 337.90 pg/mL) exhibiting higher TFEB levels compared to CU (728.12 $\:\pm\:$ 233.25 pg/mL) (*p* =.018) (Fig. 2F).

### Changes of mitophagy proteins in CSF and serum according to ATN profile

To assess if the changes in mitophagy biomarkers were influenced by the biomarker profile and severity of the biomarker load, we examined the differences in mitophagy proteins across six ATN profiles based on the positivity (+) or negativity (-) of biomarkers of amyloid beta (A), P-tau181 (T), and T-tau (N) among FTLD and AD patients *(A-T-N-*,* A-T + N-/T-N+*,* A-T + N+*,* A + T-N-*,* A + T + N-*,* A + T + N+).*

**Fig. 3:**
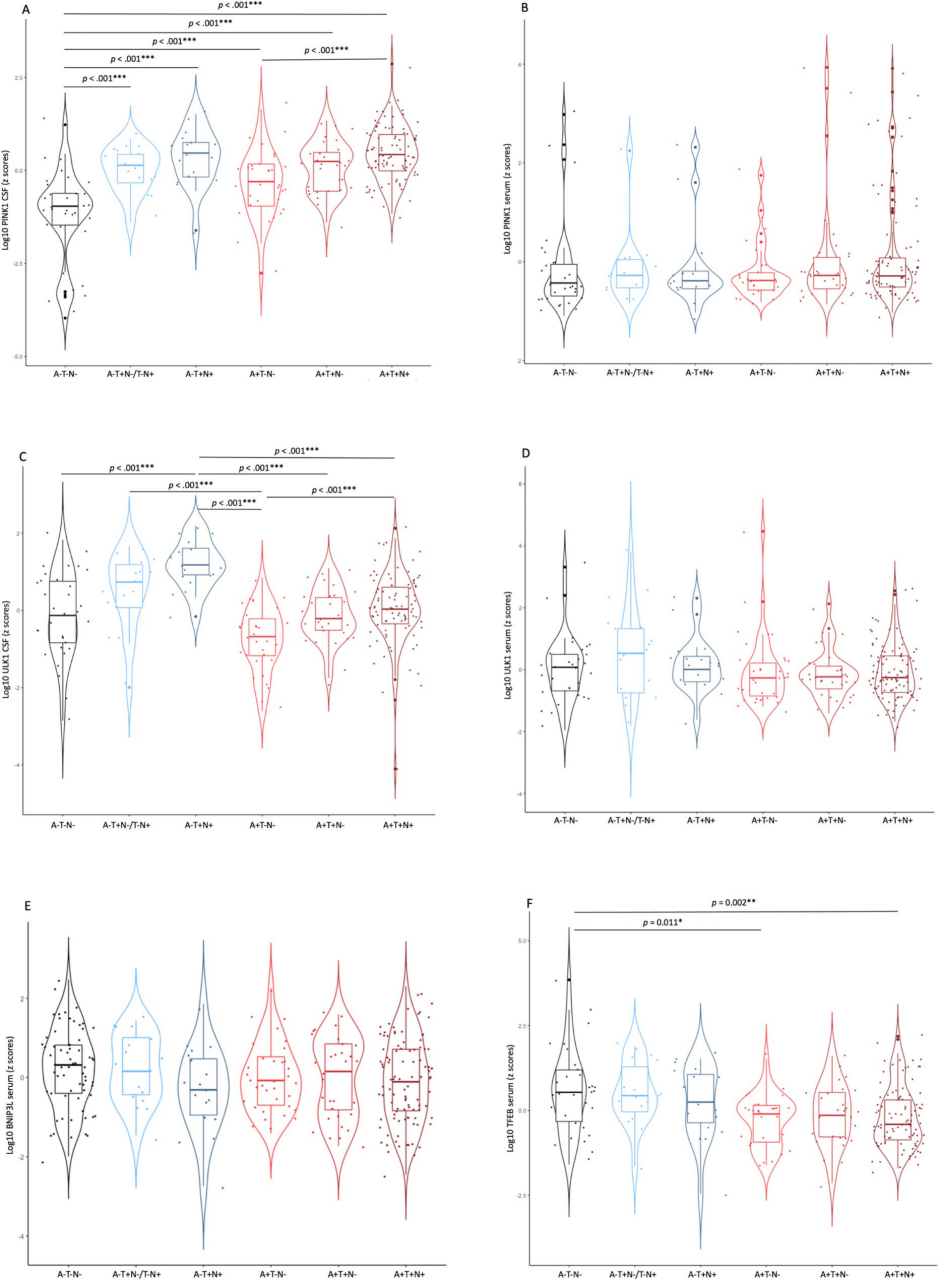
Changes of mitophagy biomarkers according to AT(N) profile. Violin plots of ANCOVA analyses (**A**-**F**) of levels of mitophagy biomarkers PINK1, ULK1, BNIP3L, and TFEB according to AT(N) profile. (**A**) CSF PINK1 showed lower levels in the A-T-N- group compared to all the other groups. It also showed lower levels in the A + T-N- group compared to the A + T + *N* + group. (**B**) Serum PINK1 levels did not show any differences among the groups. (**C**) CSF ULK1 showed higher levels in the A-T + *N* + group compared to the A-T-N-, A + T-N-, A + T + N-, and A + T + *N* + groups. It also showed lower levels in the A + T-N- group compared to the A-T + N-/T-N + and A + T + *N* + groups. (**D**) Serum ULK1 levels did not show any differences among the groups. (**E**) Serum BNIP3L levels did not show any differences among the groups. (**F**) Serum TFEB showed higher levels in the A-T-N- group compared to the A + T-N- and A + T + *N* + groups Notes: Data were adjusted for sex and age. *** *p* <.001; **p <.01; *p <.05. Abbreviations: AD, Alzheimer’s disease; FTLD, frontotemporal lobar degeneration; MCI, mild cognitive impairment; CSF, cerebrospinal fluid; CU, cognitively unimpaired

In CSF, we observed a significant difference in PINK1 levels among the AT(N) profiles (F [5] = 20.95, *p* <.001, η2 = 0.33). Post hoc analyses revealed that the A-T-N- group displayed the lowest CSF PINK1 level (0.83 $\:\pm\:$ 0.24 ng/mL), significantly differing from all the other groups: A-T + N-/T-N+ group (1.10 $\:\pm\:$ 0.17 ng/mL) (*p* =.030), A-T + N+ group (1.20 $\:\pm\:$ 0.23 ng/mL) (*p* <.001), A + T-N- group (1.01 $\:\pm\:$ 0.23 ng/mL) (*p* <.001), A + T+N- group (1.12 $\:\pm\:$ 0.20 ng/mL) (*p* <.001), and A + T+N+ group (1.24 $\:\pm\:$ 0.24 ng/mL) (*p* <.001) (Fig. 3A). CSF ULK1 levels also showed significant variation across the AT(N) groups (F [5] = 11.78, *p* <.001, η2 = 0.22). The A-T + N+ group exhibited the highest CSF ULK1 level (231.0 $\:\pm\:$ 60.5 pg/mL) and significantly differed from A-T-N- (141.5 $\:\pm\:$ 61.7 pg/mL) (*p* <.001), A + T-N- (104.1 $\:\pm\:$ 35.2 pg/mL) (*p* <.001), A + T+N- (132.6 $\:\pm\:$ 37.4 pg/mL) (*p* <.001), and A + T+N+ groups (147.7 $\:\pm\:$ 58.0 pg/mL) (*p* <.001). Additionally, the A + T-N- group displayed significantly lower levels of CSF ULK1 compared to both A-T + N-/T-N+ (180.0 $\:\pm\:$ 63.5 pg/mL) (*p* <.001) and A + T+N+ groups (*p* <.001) (Fig. 3C).

No significant differences in serum PINK1 levels (F [5] = 0.77, *p* =.570, η2 = 0.02) (Fig. 3B), serum ULK1 levels (F [5] = 1.57, *p* =.170, η2 = 0.03) (Fig. 3D) or serum BNIP3L levels (F [5] = 1.31, *p* =.261, η2 = 0.03) (Fig. 3E) were identified among the AT(N) groups. However, the AT(N) groups showed distinct levels of serum TFEB (F [5] = 4.74, *p* <.001, η2 = 0.10) with post hoc analysis revealing significantly higher levels in the A-T-N- group (1008.8 $\:\pm\:$ 672.0 pg/mL) compared to both the A + T-N- (630.2 $\:\pm\:$ 209.3 pg/mL) (*p* =.011) and A + T+N+ groups (675.0 $\:\pm\:$ 304.7 pg/mL) (*p* =.002) (Fig. 3F).

### Correlation of mitophagy markers (PINK1, ULK1, BNIP3L, and TFEB) with biomarkers of tau and neurodegeneration (P-tau181, T-tau, NfL), AT(N) framework, and cognition in FTLD continuum

We evaluated the associations between mitophagy biomarkers (PINK1, ULK1, BNIP3L, and TFEB) and biomarkers of tau and neurodegeneration (P-tau181, T-tau, NfL) in FTLD and CU individuals. NfL levels have been shown to correlate with higher disease severity, lower scores on cognitive tests, and shorter survival [32]. Additionally, differences between FTLD subtypes have been demonstrated, with more noticeable elevation in the semantic variant of primary progressive aphasia (svPPA) [33, 34]. We observed a moderate positive correlation between CSF PINK1 levels and P-tau181 (*r* =.489, *p* <.001), as well as T-tau levels (*r* =.573, *p* <.001). Similarly, CSF ULK1 levels showed moderate positive correlations with both P-tau181 and T-tau (*r* =.444, *p* <.001; *r* =.421, *p* <.001, respectively). None of the mitophagy biomarkers in serum showed significant correlations with NfL or any of the tau biomarkers (P-tau181, T-tau).

**Fig. 4:**
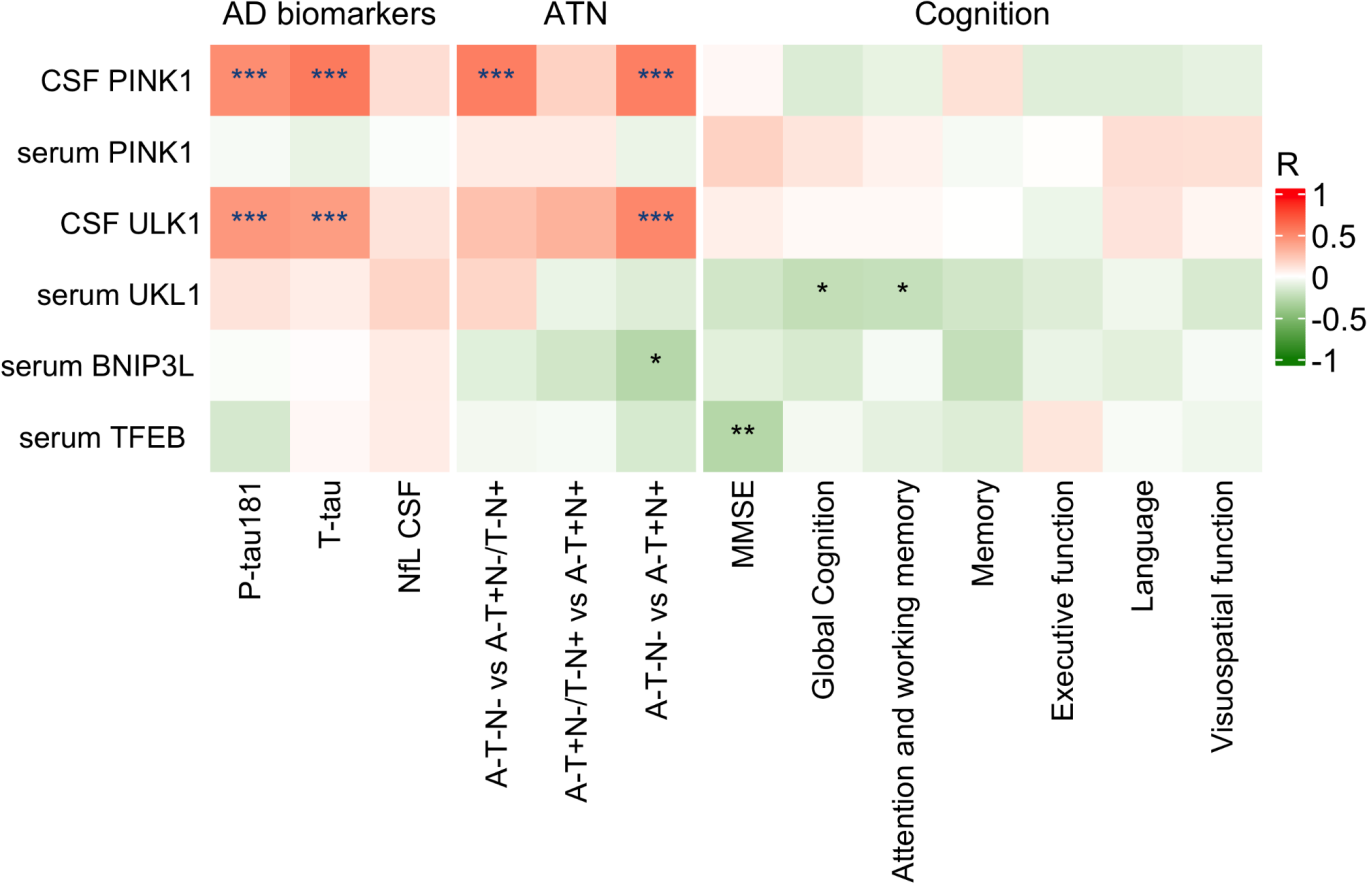
Mitophagy biomarkers vs. clinical phenotype. *, ** and *** denote *p* <.05, *p* <.01 and *p* <.001, respectively. Partial Pearson correlation for biomarkers of tau (P-tau181, T-tau) and neurodegeneration (NfL), AT(N) profile adjusted for age and sex or, in the case of cognition, for age, sex, and education. Abbreviations: CSF, cerebrospinal fluid; “A”, amyloid; “T”, tau; “N”, neurodegeneration; +, abnormal; -, normal; P-tau181, phosphorylated tau 181; T-tau, total tau; NfL, neurofilament light chain

To assess the relationship between mitophagy and the severity of biomarker pathology, we utilized the AT(N) framework. Our analyses revealed a moderate positive correlation between CSF PINK1 levels and the A-T-N- vs. A-T + N-/T-*N* + groups (*r* =.553, *p* <.001), as well as the A-T-N- vs. A-T + *N* + groups (*r* =.548, *p* <.001). (Fig. 2). Similarly, we found a moderate positive correlation between CSF ULK1 levels and the A-T-N- vs. A-T + *N* + groups (*r* =.514, *p* <.001). A weak negative correlation was observed between serum BNIP3L levels and the A-T-N- vs. A-T + *N* + groups (*r* = -.293, *p* =.048) (Fig. 4). These findings suggest that more advanced biomarker pathology is associated with higher PINK1 levels.

To investigate whether changes in mitophagy reflect cognitive decline in FTLD individuals, we correlated the levels of mitophagy biomarkers (PINK1, ULK1, BNIP3L, TFEB) with global cognition (MMSE) and cognitive composite scores. Using partial Pearson correlation controlled for sex, age and years of education, we found a negative correlation between serum TFEB levels and MMSE score (*r*=-.294, *p =*.005). Regarding other cognitive composite scores, we identified a weak negative correlation between serum ULK1 levels and the Attention and working memory domain score (*r*= -.228, *p =*.043). No other significant associations between mitophagy biomarkers and cognitive composite scores were found among FTLD and CU individuals.

### Correlation between mitophagy markers (PINK1, ULK1, BNIP3L, TFEB) in FTLD and CU groups

**Fig. 5:**
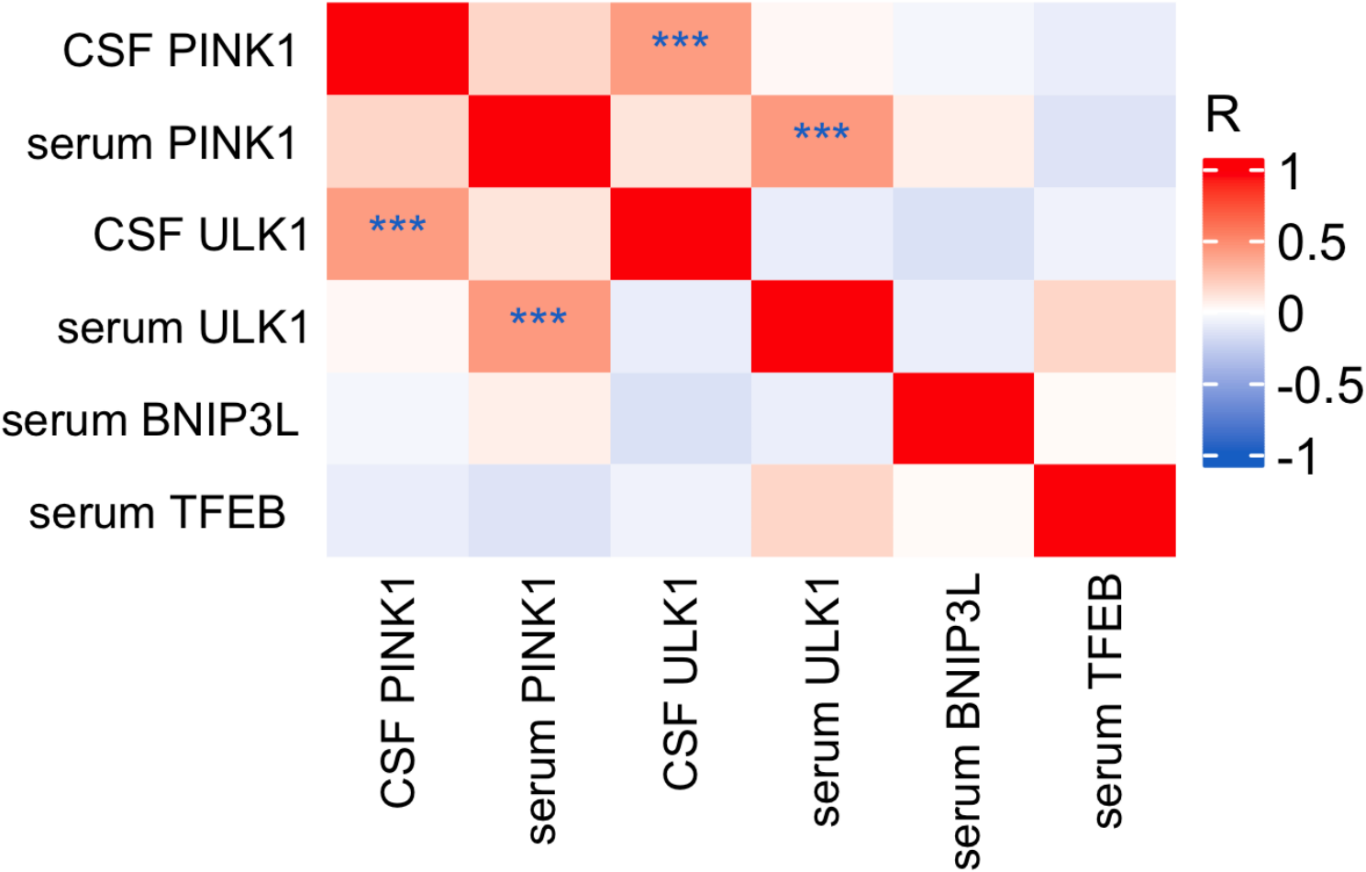
Correlation between mitophagy biomarkers in serum and CSF in FTLD. Notes: Pearson correlation, ***p <.001; **p <.01; *p <.05. Abbreviations: CSF, cerebrospinal fluid

Levels of PINK1 in CSF did not correlate with levels of PINK1 in serum (*r* =.175, *p* =.118), and levels of ULK1 in CSF did not correlate with levels of ULK1 in serum (*r*= -.095, *p* =.380). However, we identified a moderate positive correlation between CSF PINK1 and CSF ULK1 levels (*r* =.426, *p* <.001). Similarly, levels of serum PINK1 positively correlated with levels of serum ULK1 (*r* =.435, *p* <.001) (Fig. 5).

### Correlation between mitophagy markers (PINK1, ULK1, BNIP3L, TFEB) in AD and CU groups

**Fig. 6:**
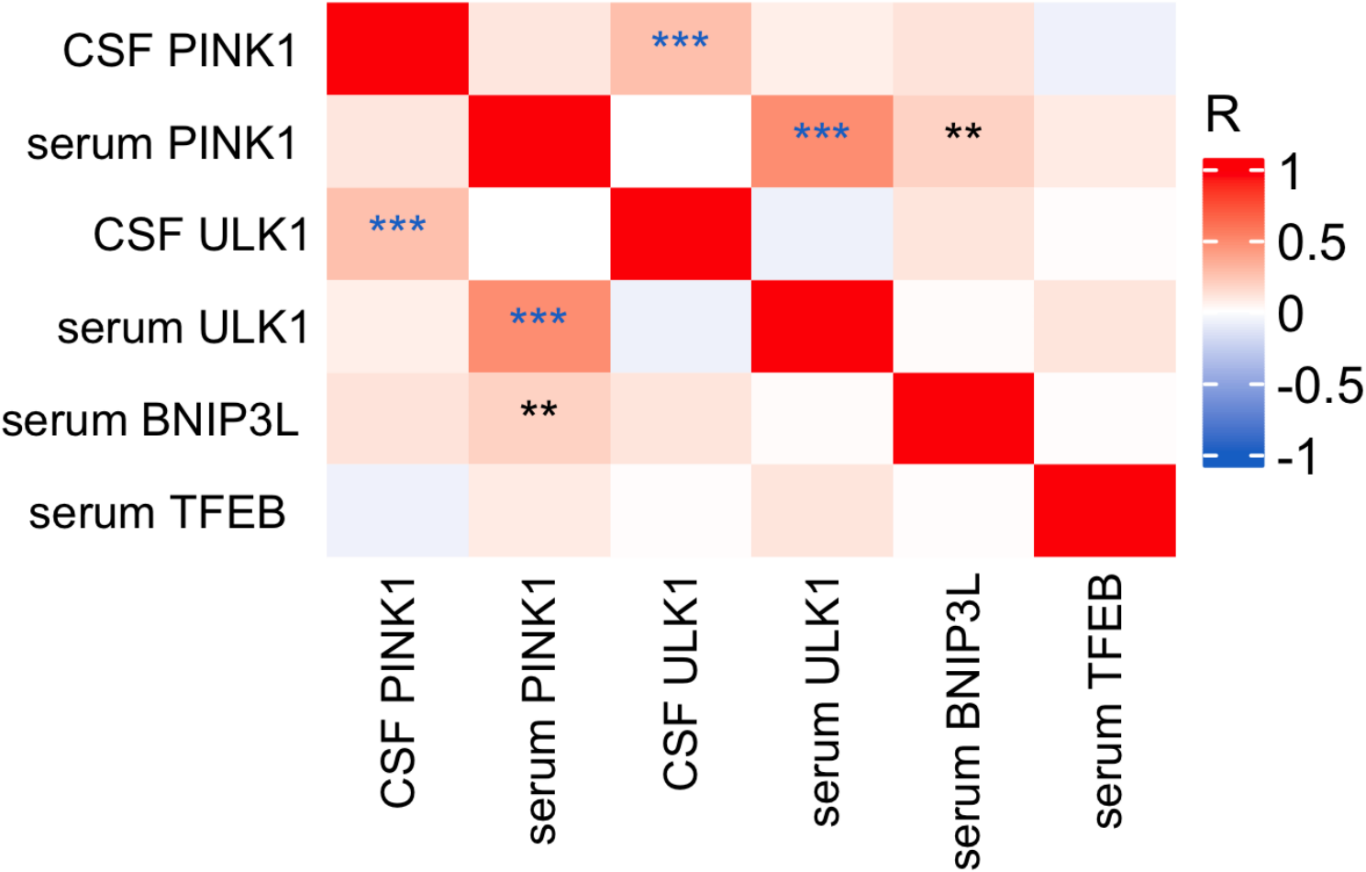
Correlation between mitophagy biomarkers in serum and CSF in AD. Notes: Pearson correlation, ***p <.001; **p <.01; *p <.05. Abbreviations: CSF, cerebrospinal fluid

Similar to FTLD, levels of PINK1 in CSF did not correlate with levels of PINK1 in serum (*r* =.105, *p* =.165), and levels of ULK1 in CSF did not correlate with levels of ULK1 in serum in AD (*r*= -.076, *p* =.286). In AD, we observed positive correlations between levels of CSF PINK1 and CSF ULK1 (*r* =.266, *p* <.001), and serum PINK1 and serum ULK1 levels (*r* =.495, *p* <.001). Additionally, levels of serum PINK1 positively correlated with levels of serum BNIP3L (*r* =.186, *p* =.008) (Fig. 6).

## Discussion

We assessed the changes in mitophagy biomarkers among patients in two clinical stages of FTLD (FTLD-MCI, FTLD dementia) and AD (AD-MCI, AD dementia), and compared them to CU. Our analyses of 308 biomarker-defined individuals show distinct alterations in CSF and serum-based mitophagy biomarkers between FTLD and AD. Specifically, we found that in CSF, levels of mitophagy activator PINK1 were significantly reduced in FTLD (both in dementia and MCI stages) compared to AD (dementia and MCI), but did not differ from CU. Similarly, serum PINK1 levels were reduced in FTLD dementia compared to AD dementia. Conversely, levels of CSF ULK1, the main autophagy initiator, were increased in FTLD-MCI compared to AD dementia patients, and serum ULK1 levels were increased in FTLD dementia relative to AD-MCI and CU individuals. Additionally, serum TFEB levels, a master regulator of lysosomal function and autophagy, were significantly elevated in FTLD dementia compared to AD dementia, AD-MCI, and CU controls, as well as higher levels in FTLD-MCI compared to AD dementia. These findings underscore specific differences in mitophagy regulation between FTLD and AD, suggesting that these neurodegenerative diseases may affect the cellular waste disposal system through potentially distinct mechanisms, as reflected by the differential mitophagy biomarker profiles.

PINK1 is a serine/threonine protein kinase that stabilizes on the outer mitochondrial membrane upon mitochondrial stress or damage to phosphorylate its substrates and initiate mitophagy [35]. In our study, we observed significantly lower CSF and serum PINK1 levels in FTLD compared to AD, but unchanged compared to CU. Previous findings from a cellular model and iPSC-derived cortical neurons from FTLD patients with *GRN* mutations (FTLD-TDP) suggested that PGRN influences PINK1 stability and/or activity, potentially contributing to mitophagy dysfunction [36]. This hypothesis is supported by data showing reduced PINK1 accumulation and decreased levels of mitochondrial phospho-ubiquitin following pharmacological induction of mitophagy in *GRN* mutation patient-derived iPSC [34]. Additionally, inhibition of TDP-43 phosphorylation significantly restored mitochondrial function in *GRN* knockdown cells, implicating TDP-43 pathology as a critical contributor to the mitochondrial dysfunction observed in PGRN-deficient cells [36]. Accordingly, research on cell cultures and TDP-43 transgenic mice has shown that an excess of TDP-43 impairs proteasomal activity and selectively impedes PINK1 protein turnover, causing the accumulation of PINK1 protein aggregates in diseased neurons, resulting in compromised mitochondrial activity [37]. Although results from basic research have suggested an interaction between FTLD pathology and PINK1, we did not find significant differences in PINK1 levels in FTLD compared to CU. The reason for this discrepancy is not clear. One possible explanation is that animal models may not accurately reflect the complexity of changes occurring in humans. In FTLD, the lower levels of PINK1 compared to AD might reflect a distinct mechanism of mitophagy impairment in this disease group. This could be attributed to the differential impact of PGRN and TDP-43 on mitochondrial function in FTLD, contrasting with the upregulation of mitophagy markers observed in AD.

ULK1 is a key regulator of autophagy and mitophagy, part of a protein complex essential for autophagy induction, controlling the initial stages of autophagosome formation [38]. In our study, we found increased CSF ULK1 levels in FTLD-MCI compared to AD dementia and AD-MCI, and increased serum ULK1 in FTLD dementia compared to CU and AD-MCI. This might indicate a compensatory response to impaired autophagy in the prodromal stage of FTLD, as neurons attempt to mitigate cellular stress and maintain homeostasis. Increased autophagic activity was also reported in the absence of C9orf72 [39, 40]. However, despite this overall increase in autophagic flux, the initiation of autophagy appeared to be compromised in the absence of C9orf72 [39, 41]. These findings are in contrast with AD, where autophagy pathways, including ULK1 signaling, might be more severely disrupted, leading to lower ULK1 levels. The differences in ULK1 expression between FTLD and AD suggest that, while autophagic dysfunction appears to contribute to the development and progression of both diseases, the underlying mechanisms and cellular responses may vary.

In FTLD, increased ULK1 levels might indicate a compensatory response to impaired autophagy as neurons attempt to mitigate cellular stress and maintain homeostasis. Conversely, in AD, more severe disruptions in autophagy pathways, including ULK1 signaling, may lead to lower ULK1 levels. This suggests that autophagy is not only involved in the pathology of these diseases but may also actively contribute to their progression through different mechanisms.”

TFEB is a major transcription factor crucial for autophagy and lysosomal biogenesis, playing a role in the final autolysosomal degradation. It has previously been shown that both C9orf72 and TDP-43 are key regulators of TFEB activity [42]. Loss of these proteins led to decreased mTOR activity and increased nuclear translocation of TFEB, enhancing autophagy, lysosomal biogenesis, and lysosomal protein (LAMP1, LAMP2) expression [40, 43]. In addition, loss of TDP-43 impaired the fusion of autophagosomes with lysosomes. The combination of these two processes led to an accumulation of immature autophagic vesicles and overwhelmed autophagy-lysosome function [40]. Increased TFEB activity and expression were also found in response to tau pathology in the frontal cortex of subjects with FTLD and in transgenic mouse models. Enhanced TFEB activity improved tau uptake and lysosomal function in cultured primary astrocytes from wild-type mice, suggesting a compensatory mechanism in response to tau accumulation [44]. Also, *MAPT* p.R406W mutation in FTLD-tau patients disrupted lysosomal biogenesis and autophagic function, resulting in elevated TFEB expression in the insular cortex [42]. These findings from FTLD subtypes in animal and cell models contrast with previous findings in AD, where TFEB was found to be reduced [43, 44]. Our findings of elevated serum TFEB levels in FTLD patients compared to AD as well as CU individuals corroborate this notion. Collectively, these findings suggest that while TFEB activation in FTLD may act as a cellular defense mechanism, it is not sufficient to fully compensate for the underlying disease-mediated dysfunction of autophagy.

Since each clinical phenotype of FTLD (bvFTLD, svPPA, nvPPA) is known to be associated with distinct underlying protein pathologies, we sought to investigate whether the observed differences in mitophagy biomarkers between FTLD and CU individuals might be driven by a single clinically defined FTLD subtype. It is known that bvFTLD is associated with TDP-43 pathology (FTLD-TDP) in around 50% of cases and with tau pathology (FTLD-tau) in 40% of cases, while svPPA generally shows TDP-43 inclusions and in nvPPA, tau-positive inclusions are most commonly found [45]. However, it is crucial to acknowledge that the precise molecular pathology underlying these clinical subtypes cannot be definitively determined without neuropathological examination of brain tissue, which remains the gold standard for confirming specific protein inclusions such as TDP-43 or tau. When examining the mitophagy biomarker levels among different FTLD subtypes and comparing them to CU individuals, we found that the mitophagy biomarker changes were more prone in bvFTLD and nvPPA clinical subtypes. These subtypes (bvFTLD, nvPPA) are associated with various pathological proteins, but they are more commonly linked to tau pathology (FTLD-tau), whereas svPPA is typically linked to TDP-43 pathology [6, 7]. Specifically, bvFTLD patients displayed lower CSF PINK1 levels compared to controls and a trend towards lower levels compared to svPPA. Additionally, serum TFEB levels were significantly elevated in nvPPA compared to controls. However, it is important to note that the majority of the mitophagy biomarkers examined in this study did not show significant differences between FTLD subtypes and CU individuals. Given these findings, we further explored the mitophagy biomarker profile in relation to the ATN classification system, which provides a comprehensive assessment of biomarker pathology [17].

To determine if the changes in mitophagy biomarkers were affected by the biomarker profile and severity, we analyzed the differences in mitophagy proteins across six ATN profiles, classified by the positive (+) or negative (-) status of P-tau181 (T), and T-tau (N) in both AD patients (A+) and FTLD (A-) patients. The analysis of mitophagy biomarkers according to the ATN profile revealed significant differences in CSF PINK1, ULK1, and serum TFEB levels. Notably, CSF PINK1 and ULK1 levels were markedly elevated in groups with more advanced pathology in both FTLD (A-T + N+) and AD (A + T + N+) compared to the groups with negative P-tau181 and T-tau (A-T-N-, A + T-N-). This finding aligns with our observation of a positive correlation between these same mitophagy markers and CSF tau (P-tau181, T-tau) in FTLD individuals, suggesting a potential link between mitophagy dysfunction and tau pathology. The findings indicate that as neurodegeneration progresses, as reflected by increasing biomarker positivity within the ATN classification, neurodegeneration affects mitophagy biomarker profiles similarly in both A- (FTLD) and A+ (AD) individuals. However, due to differences in disease mechanisms, baseline levels of these biomarkers remain consistently higher in FTLD patients compared to AD patients in similar TN categories.

Our study did not identify an association between mitophagy biomarkers and specific cognitive domains in FTLD patients, with the exception of a negative correlation between serum TFEB levels and global cognitive function (MMSE). This lack of widespread correlation might be attributed to the well-established heterogeneity in FTLD [45]. Unlike AD, which primarily affects memory, FTLD manifests with a diverse spectrum of cognitive impairments depending on the underlying proteinopathy and affected brain regions [46]. This variability in clinical presentation within FTLD could potentially mask subtle associations between mitophagy dysfunction and specific cognitive domains.

To understand the relationship between individual mitophagy biomarkers, we examined their mutual correlations. We found a positive correlation between CSF PINK1 and CSF ULK1 levels, and between serum PINK1 and serum ULK1 levels in both disease groups. PINK1 and ULK1 are both considered a key activators of mitophagy and autophagy, respectively. These findings indicate potential coordinated activity of PINK1 and ULK1, irrespective of the underlying neurodegenerative disease state [47]. Interestingly, in line with our previous finding in AD, we did not identify a correlation between PINK1 levels in CSF and serum in FTLD [4]. Also, ULK1 levels did not correlate in CSF and serum in both FTLD and AD. This lack of correlation might be due to the fact that peripheral tissues, such as muscle, liver, and kidney, also express PINK1 and ULK1. The proteins produced in these peripheral tissues can enter the bloodstream and contribute to the overall serum levels, thereby potentially masking or diluting the specific changes in PINK1 and ULK1 concentrations that occur within the central nervous system.

Limitations of our study include the absence of neuropathological confirmation, which prevents definitive attribution of mitophagy marker alterations to specific FTLD molecular subtypes. Future studies integrating neuropathological data will be critical for validating these findings. The relatively small number of patients in certain groups, may limit the generalizability of our findings. Nonetheless, to the best of our knowledge, this is the first study to comprehensively investigate CSF and serum-based mitophagy biomarkers in FTLD. Strengths of our study include a well-defined cohort of clinically and biomarker-defined FTLD patients spanning the wide clinical spectrum of the disease. Additionally, our study benefits from a direct comparison with AD patients for differential analysis, allowing us to identify potential disease-specific alterations in mitophagy biomarkers.

## Conclusions

Our study provides compelling evidence of distinct alterations in autophagy/mitophagy biomarkers between FTLD and AD, indicating that these neurodegenerative diseases may affect the cellular waste disposal system through different pathways. Specifically, ULK1 and TFEB levels were elevated in FTLD compared to AD dementia patients and CU individuals. The increased ULK1 levels observed in FTLD-MCI may represent a compensatory response to autophagy impairment in the early stages of FTLD. The elevated serum TFEB levels observed in FTLD support the notion of enhanced autophagic and lysosomal activity as a compensatory response to neurodegeneration. Overall, the identified alterations in FTLD were more associated with proteins associated with general autophagy function. In contrast to AD, no changes were found in the mitophagy-specific protein PINK1. Further research is required to elucidate the pathological mechanisms underlying these differences, which could facilitate the development of targeted therapeutic strategies for each condition. Future studies should include larger sample sizes, longitudinal design, neuropathological confirmation, and comprehensive genetic subtyping to validate and expand upon our findings and to better understand the role of specific neuropathological subtypes and genetic mutations in shaping mitophagy pathways in FTLD.

## Data Availability

The datasets used and/or analysed during the current study available from the corresponding author on reasonable request.

## Abbreviations

Aβ: Amyloid beta
AD: Alzheimer‘s disease
BNIP3L: BCL2/adenovirus E1B 19 kDa protein-interacting protein 3-like
bvFTD: Behavioral variant of frontotemporal dementia
CSF: Cerebrospinal fluid
CU: Cognitively unimpaired
FTLD: Frontotemporal lobar degeneration
MCI: Mild cognitive impairment
MMSE: Mini-Mental State Examination
NfL: Neurofilament light chain
nvPPA: Nonfluent variant of primary progressive aphasia
P-tau181: Phosphorylated tau at threonine 181
PINK1: PTEN- induced Kinase 1
svPPA: Semantic variant of primary progressive aphasia
T-tau: Total tau
TDP-43: TAR DNA binding protein 43
TFEB: Transcription factor EB
ULK1: Unc-51-like kinase 1
